# Ambulante spezialfachärztliche Versorgung (ASV) in der Humangenetik

**DOI:** 10.1515/medgen-2025-2001

**Published:** 2025-02-12

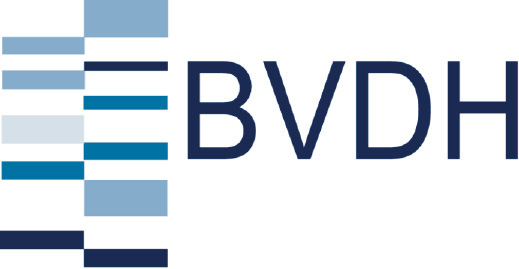



Die ASV ist ein Versorgungskonzept für Patienten mit komplexen Erkrankungen, die einer interdisziplinäre Zusammenarbeit von spezialisierten Ärzten verschiedener Fachrichtungen bedürfen, sowohl aus Krankenhäusern als auch aus dem niedergelassenen Bereich [2, 4]. In der ASV ist die Humangenetik zwar nicht als eigenständiger Schwerpunkt definiert, aber sie ist in vielen Indikationsbereichen von großer Bedeutung.

**Rechtliche Grundlagen und Entwicklung der ASV:** Die ASV wurde 2012 als Weiterentwicklung der früheren ambulanten Behandlung im Krankenhaus (ABK) eingeführt [2]. Der Gemeinsame Bundesausschuss (G-BA) legt in der ASV-Richtlinie die generellen und erkrankungsspezifischen Anforderungen fest [2].

**Berechtigte Leistungserbringer:** An der ASV können sowohl Vertragsärzte als auch nach § 108 SGB V zugelassene Krankenhäuser teilnehmen.

**Interdisziplinäre Teams:** Die ASV-Teams bestehen aus einer Teamleitung und Kernteammitgliedern sowie bei Bedarf hinzuzuziehenden Fachärzten [1]. Die personellen Anforderungen können auch durch vertraglich vereinbarte Kooperationen erfüllt werden [1].

**Indikationen und Leistungsbereiche entsprechend GBA:**

**Onkologische Erkrankungen:** Gastrointestinale-, Gynäkologische-, Urologische-, Lungen- und Thorax, Haut-, Kopf- oder Halstumore
**Seltene Erkrankungen:** Marfan-Syndrom, Mukoviszidose, Morbus Wilson, Seltene Lebererkrankungen
**Chronische Erkrankungen:** Tuberkulose und atypische Mykobakteriose, Pulmonale Hypertonie, Rheumatologische Erkrankungen bei Erwachsenen und Kindern/Jugendlichen, Sarkoidose
**Weitere Indikationen:** Hämophilie, Epilepsie, seltene neuromuskuläre Erkrankungen, multiple Sklerose, Parkinson-Syndrom, Amyotrophe Lateralsklerose

**Abrechnung und Vergütung:** Sie erfolgt nach einheitlichen Vorgaben, die in der ASV-Vereinbarung (ASV-AV) geregelt sind [3]. Wesentliche Punkte sind:

Getrennte, aber inhaltlich gleiche Abrechnungsverfahren für Vertragsärzte und Krankenhäuser
Einführung einer Teamnummer zur eindeutigen Identifikation des interdisziplinären Teams
Verwendung spezieller Vordrucke für die ASV-Abrechnung
Extrabudgetäre Vergütung zu festen Preisen ohne Mengenbegrenzung [3, 4]

**Integration der Humangenetik in ASV-Teams:** Bei vielen Indikationen der ASV ist die Einbindung humangenetischer Expertise in das interdisziplinäre Team vorgesehen oder empfohlen. Dies kann auf verschiedene Weise erfolgen:

1. Als fester Bestandteil des Kernteams: Bei einigen seltenen Erkrankungen ist ein Facharzt für Humangenetik explizit als Mitglied des Kernteams vorgesehen.
2. Als hinzuzuziehender Facharzt: In vielen Fällen wird die humangenetische Expertise bei Bedarf hinzugezogen, etwa zur Durchführung spezieller diagnostischer Tests oder zur genetischen Beratung.
3. In Form von Kooperationen: ASV-Teams können mit humangenetischen Zentren oder Praxen kooperieren, um die erforderliche Expertise sicherzustellen.

**Herausforderungen und Zukunftsperspektiven:** Die ASV befindet sich noch in der Entwicklung, und es werden kontinuierlich neue Indikationen hinzugefügt. Herausforderungen bestehen in der praktischen Umsetzung der sektorenübergreifenden Zusammenarbeit und der Integration neuer diagnostischer und therapeutischer Möglichkeiten, insbesondere im Bereich der Genetik.

**Zukunftsperspektive der Humangenetik in der ASV:** Die Bedeutung der Humangentik in der ASV wird voraussichtlich weiter zunehmen. Einige Trends und Entwicklungen, die diese Prognose unterstützen, sind: Fortschritte in der Genomsequenzierung, Entwicklung der personalisierten Medizin, der Pharmakogenetik und der präventiven Genetik.

**Fazit und Theorie:** Durch die Förderung der interdisziplinären und sektorenübergreifenden Zusammenarbeit bietet die ASV das Potenzial, Behandlungsqualität und -effizienz zu steigern. Die Humangenetik spielt in diesem Kontext eine zunehmend wichtige Rolle. Mit dem wachsenden Verständnis genetischer Grundlagen von Krankheiten und der Entwicklung personalisierter Therapieansätze wird ihre Bedeutung in der ASV voraussichtlich weiter zunehmen. Alle im Rahmen eines ASV erbrachten Leistungen werden voll vergütet und entlasten damit das Budget der Humangentik, die Abrechnung erfolgt über die jeweils zuständige KV.

**Praxis:** Es ist allerdings festzuhalten, dass insbesondere eine Krankenkasse häufig für mehrere Jahre rückwirkend Rückforderungen formuliert. Streng nach dem Motto: Der Patient ist versorgt, das Geld wird zurückgefordert. Es ist daher grundsätzlich darauf zu achten, dass die Kostenübernahme im Vorfeld verbindlich geklärt wird.

Der Vorstand des BVDH
